# *Ocruranus*–*Eohalobia* Sclerites from the Cambrian Stage 2 Yanjiahe Formation in South China: Scleritome Reconstruction and Zoological Affinity

**DOI:** 10.3390/biology11111648

**Published:** 2022-11-11

**Authors:** Zuchen Song, Junfeng Guo, Bing Pan, Yaqin Qiang, Guoxiang Li, Jiaxin Peng, Jie Sun, Jian Han

**Affiliations:** 1 Key Laboratory of Western Mineral Resources and Geological Engineering, Ministry of Education, School of Earth Science and Resources, Chang’an University, Xi’an 710054, China; 2 State Key Laboratory of Palaeobiology and Stratigraphy, Nanjing Institute of Geology and Palaeontology and Center for Excellence in Life and Paleoenvironment, Chinese Academy of Sciences, Nanjing 210008, China; 3 Shaanxi Key Laboratory of Early Life and Environments, Department of Geology, Northwest University, Xi’an 710069, China

**Keywords:** *Ocruranus*–*Eohalobia* group, Polyplacophora, muscle attachment zone, Cambrian Stage 2, South China

## Abstract

**Simple Summary:**

New materials of *Ocruranus* and *Eohalobia* sclerites are found in Member 5 of the Yanjiahe Formation (Cambrian Stage 2). The extended and upfolded field on *Eohalobia* exhibiting dense wrinkles is most likely a weakly mineralized structure that joins the valves. The distribution of muscle attachment zones of the *Ocruranus*–*Eohalobia* group corresponds with that in modern chitons, which provides strong evidence for assigning them to the Polyplacophora. Furthermore, comparing the butterfly-shaped region on a unique specimen of *Eohalobia* with the sub-apical field on *Ocruranus* suggests that the original scleritome may consist of only *Ocruranus* and *Eohalobia* sclerites. The *Ocruranus*–*Eohalobia* group previously discovered from the Zhongyicun Member (Fortunian) in eastern Yunnan may have three types of plates, which is different from the reconstruction here from the Yanjiahe Formation (Stage 2), where only two plates are included. However, it is inferred that such differences might be due to the morphologic evolution (the reduction of the number of plates from three to two from the Fortunian to Stage 2) or due to other factors (geographical, biological, etc.). This paper provides a new insight into reconstructing the original configuration of the scleritome of the *Ocruranus*–*Eohalobia* group.

**Abstract:**

The isolated sclerites of the *Ocruranus* and *Eohalobia* group are abundant among the early Cambrian small shelly fossil assemblages, which were recently assigned to the same scleritome as an early member of the polyplacophoran (chiton) stem lineage. However, the scleritome reconstruction and zoological affinities of these sclerites are still controversial due to the lack of exceptionally preserved articulated specimens with in-situ sclerites. Herein, we report new specimens of *Ocruranus* and *Eohalobia* sclerites from Member 5 of the Yanjiahe Formation, which provide new insights into the reconstruction of the original scleritome. The *Eohalobia* sclerites from the Yanjiahe Formation have an extended and upfolded proximal field with dense wrinkles, which seems to be a weakly mineralized structure and acted as a joint with another sclerite, *Ocruranus*. Comparing the butterfly-shaped proximal field on a unique sclerite of *Eohalobia* with the sub-apical field on *Ocruranus* sclerites suggests that the original scleritome of this group may consist of only two types of sclerites: the *Ocruranus*-type and the *Eohalobia*-type. The polygonal structure on the internal mold of *Eohalobia* sclerites is interpreted herein as the muscle attachment zone; their distribution corresponds well with that of the modern chitons, which provides strong evidence to support the close relationship between the *Ocruranus*–*Eohalobia* group and the Polyplacophora.

## 1. Introduction

The small shelly fossils (SSFs), represented by spicules, shells, tubes, and diverse disarticulated sclerites or complete scleritomes, are a wealth of micro-sized skeletal fossils that belong to various animal phyla. The SSFs widely occurred in the pre-trilobite Cambrian strata (Fortunian and Stage 2) in South China, Siberia, Mongolia, Australia, and France [1,2,3,4,5,6,7]. They provide considerable information on the first appearance of the major phyla of the animal kingdom and shed light on the nature and chronology of the animal radiation [8,9,10,11]. SSF assemblages are particularly rich and diverse through the early Cambrian of China during the Ediacaran–Cambrian transition [1,3,4,12,13,14,15,16].

SSF assemblages contain a great variety of isolated elements, such as sclerites, spines, plates, and scalids, that used to be part of scleritomes and cuticular exoskeletons. One of the most challenging tasks for scientists is to reconstruct these scleritomes and to establish a coherent taxonomy knowing that one type of sclerite may have several morphotypes that have often received different names. One representative case is the ‘*Ocruranus*–*Eohalobia*’ group, a term initially proposed by Qian and Bengtson [12] to highlight a close relationship between these two genera (two types of sclerites). *Ocruranus* designates a particular type of sclerite characterized by a cap-shaped shell with a recessed sub-apical field, which was widely distributed among different palaeocontinents in the early Cambrian, such as South China (eastern Yunnan and western Hubei provinces) [15,17,18,19,20,21,22], France [5,23], Greenland [24], and Mongolia [25]. Being similar in age to *Ocruranus*, *Eohalobia,* which has predominantly been reported in South China (eastern Yunnan and western Hubei provinces), has an elongated shell with an arched proximal (apical) margin and rounded distal margin. Qian and Bengtson [12] conducted a detailed taxonomic revision and referred to them as the ‘*Ocruranus*–*Eohalobia* group’, based on their co-occurrences and similar morphologies (i.e., subapical field and a triangular proximal part with a convex central part and/or re-entrant). However, their taxonomic affinities are still very uncertain. *Ocruranus* was previously assigned to a paterinid brachiopod [19,20], but is currently more commonly considered as a stem member of polyplacophorans (chitons) together with *Eohalobia* [21,26,27]. *Eohalobia* was also initially considered to have various zoological affinities (e.g., bivalves and tommotiids) [18,20]. Similarly, the ‘*Ocruranus*–*Eohalobia* group’ was variously interpreted as parts of a coeloscleritophoran scleritome, such as an halkieriid [28] and *Maikhanella* [29,30], or a possible early mollusk [24]. Vendrasco et al. [26] speculated that *Gotlandochiton*? *minimus* Yu [21] was a third type of sclerite in the ‘*Ocruranus*–*Eohalobia*’ group, and considered that *Ocruranus* sclerites, *Gotlandochiton*? *minimus* sclerites, and *Eohalobia* sclerites were parts of the same scleritome of an organism belonging to the stem-lineage of chitons. However, the detailed jointed mode of the shell plates, the number of valves, and the anterior-posterior orientation of the ‘*Ocruranus*–*Eohalobia* group’ remain controversial.

Recently, we recovered *Ocruranus*–*Eohalobia* sclerites from the Yanjiahe Formation in the Three Gorges area of South China. Based on the new material and previous literature, this study aims to reinvestigate the morphological characteristics of *Ocruranus* and *Eohalobia* sclerites, and the shell microstructures, as well as the distributions of the muscle attachment zones in *Eohalobia*. This study also discusses the scleritome reconstruction of the *Ocruranus*–*Eohalobia* group and explores its zoological affinity, which may be assigned to a stem group aculiferans placed in a basal clade of the extant Mollusca [31].

## 2. Geological Setting, Materials and Methods

**Figure 1:**
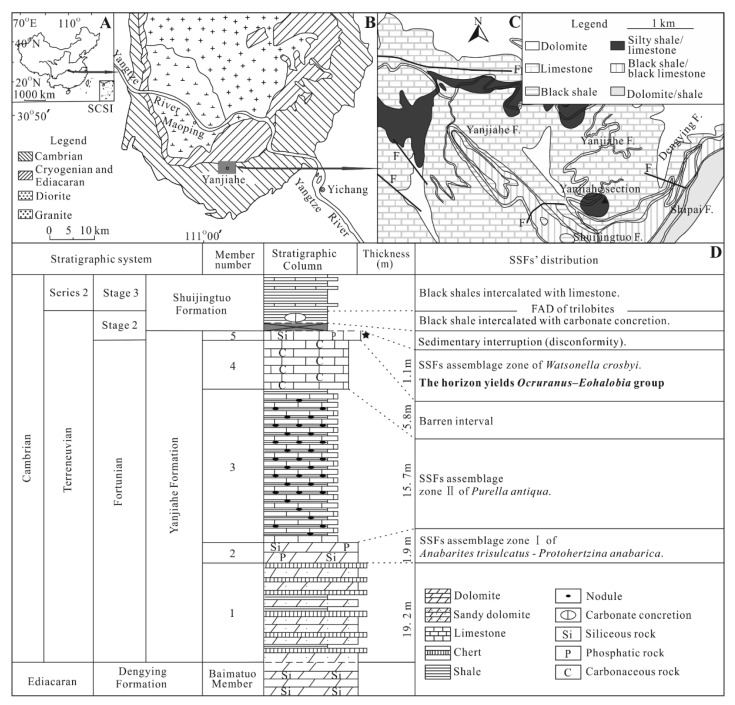
Location and stratigraphy of the Terreneuvian Yanjiahe Formation in the Three Gorges area, Hubei Province, China (modified from [14]). (**A**) Sketch map of the People’s Republic of China, showing the position of the collecting locality in Hubei Province. (**B**) Simplified geological map of the Three Gorges area, Hubei Province, showing the outcrops of the Cambrian strata (gray box in the Yanjiahe area denotes area enlarged for additional details). (**C**) Detailed geological map of the Yanjiahe area, showing the outcrops of the Yanjiahe Formation. (**D**) Stratigraphic sequence of the Yanjiahe Biota in the Yanjiahe section, Yangtze Gorges area, indicating the horizon where specimens of *Ocruranus* and *Eohalobia* sclerites were collected.

All studied specimens of *Ocruranus*–*Eohalobia* were collected from Member 5 of the well-studied Yanjiahe Formation [13,14,22,32,33] at the Yanjiahe section near Yichang city (Figure 1). The Yanjiahe Formation is particularly well-exposed near Yanjiahe village, Hubei Province, and is subdivided into five members based on the lithology (originally described as five beds) [13]. Member 5 of the Yanjiahe Formation is mainly composed of phosphatic limestone and belongs to the *Watsonella crosbyi* Assemblage Zone of South China, which is correlated to Cambrian Stage 2, the thickness of which is about 1.1 m (Figure 1D).

**Figure 2:**
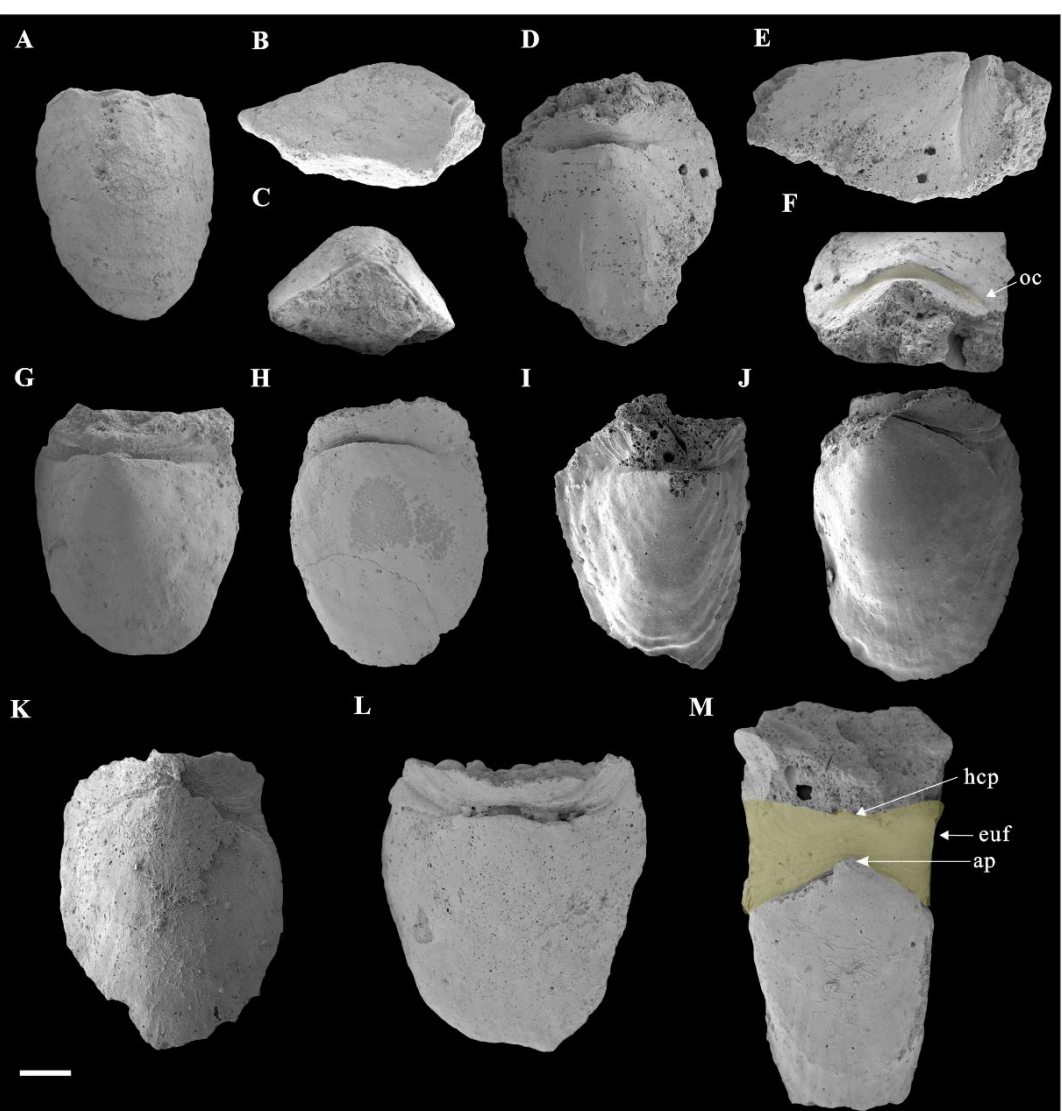
*Eohalobia* sclerites, from Member 5 of the Yanjiahe Formation in Yanjiahe section (*Watsonella crosbyi* Assemblage Zone). (**A**–**C**), (**D**–**F**) Internal molds, CUBar228–2, CUBar228–5, respectively. (**G**–**M**) Internal molds, CUBar110–7, CUBar212–1, CUBar101–1, CUBar101–2, CUBar40–1, CUBar212–3, CUBar212–2, respectively. (**A**,**D**,**G**–**M**) Dorsal view. (**B**,**E**) Lateral view. (**C**,**F**) Proximal view. Colored areas show oblique cavity and butterfly-shaped extended field. Scale bar equals 500 μm. Abbreviations: ap = apex of shell; euf = the extended and upfolded field; hcp = high-arched central part; oc = oblique cavity.

**Figure 3:**
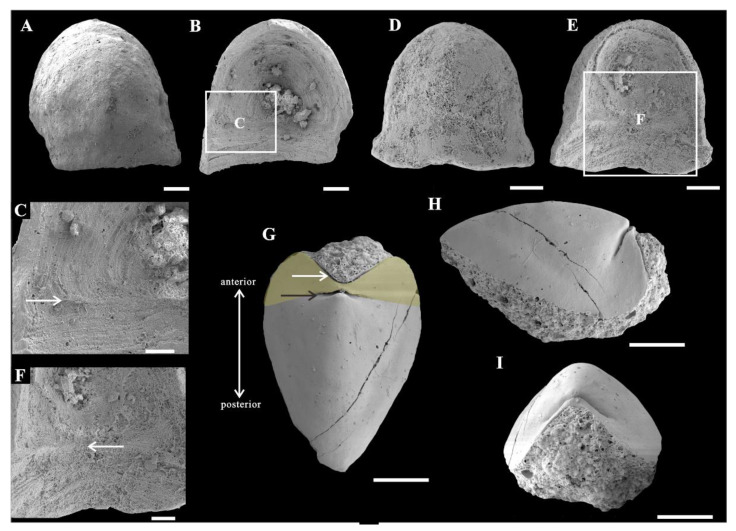
*Ocruranus* sclerites and *Eohalobia* sclerite from Member 5 of the Yanjiahe Formation. (**A**–**C**) Cast of *Ocruranus* sclerite, CUBar169–16; (**A**) exterior view; (**B**) interior view; (**C**) magnification of (**B**), showing dense wrinkles and transverse ridge. (**D**–**F**) Cast of *Ocruranus* sclerite, CURBar13–5; (**D**) exterior view; (**E**) interior view; (**F**) magnification of (**E**), showing dense wrinkles and transverse ridge. (**G**–**I**) Internal mold of *Eohalobia* sclerite, CUBar228–7; (**G**) dorsal view; (**H**) lateral view; (**I**) proximal view. White arrows show transverse ridge on inner surface of *Ocruranus* sclerites in (**C**,**F**) and V-shaped furrow on *Eohalobia* sclerite in (**G**). Black arrow shows transverse furrow on *Eohalobia* sclerite. Colored area shows butterfly shape. Scale bar: (**A**,**B**,**D**,**E**) 200 μm; (**C**,**F**) 100 μm; (**G**–**I**) 500 μm.

Rock samples were treated with buffers of approximately 10% acetic acid under a fume hood to retrieve acid-resistant microfossils. All of the selected specimens (Figure 2 and Figure 3) were coated with gold and then imaged using an FEI Quanta 650 scanning electron microscope (SEM) at Chang’an University. A total number of 28 *Ocruranus* sclerites and 44 *Eohalobia* specimens were collected and are housed in the paleontological collections of Chang’an University (CU) in Xi’an, China. ‘Bar’ represents aluminum stub. ‘RBar’ represents rotational aluminum stub.

## 3. Results

### 3.1. Morphology of Ocruranus and Eohalobia Sclerites

The new material of *Ocruranus* sclerites is preserved by secondary phosphatization and retrieved from Member 5 of the Yanjiahe Formation. All specimens are approximately 1 mm in width and 1.5 mm in length. These cap-like specimens have a nearly rounded outline and a straight proximal edge from a dorsal view and an obtuse round apex (Figure 3A,D). The sub-apical field showing a butterfly shape is extended and forms an obtuse angle; the outer surface of the cast is smooth (Figure 3A,D). The inner surface of these specimens exhibits dense wrinkles, and the initiation of the extended sub-apical field is a prominent transverse ridge (Figure 3B,C,E,F).

**Figure 4:**
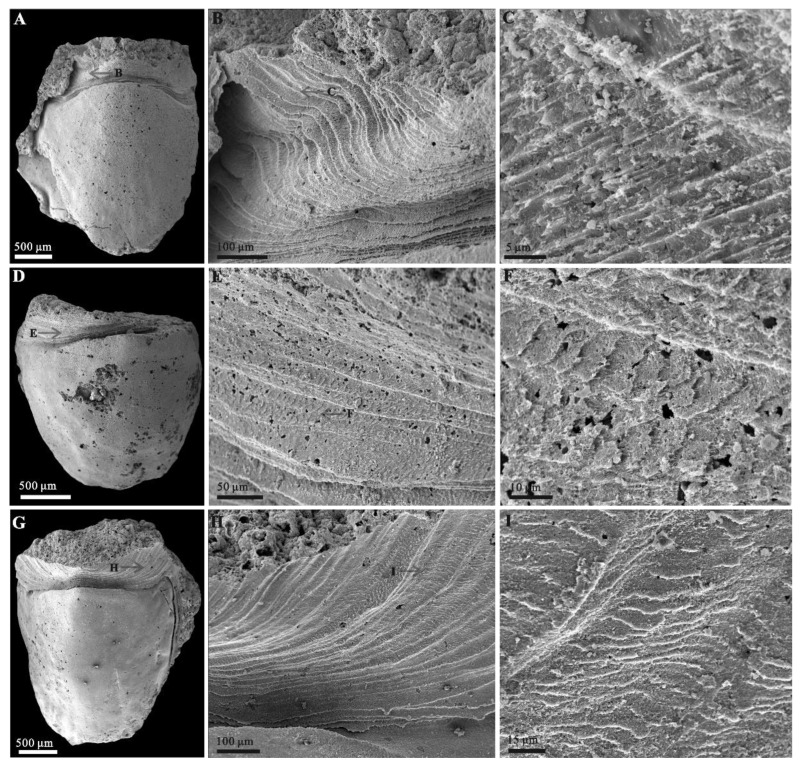
Wrinkles on the extended and upfolded field of *Eohalobia* sclerites. (**A**–**C**), (**D**–**F**), (**G**–**I**) Internal molds; CUBar126–12, CUBar126–7, CUBar70–25, respectively. (**B**,**E**,**H**) Magnification of (**A**,**D**,**G**), respectively, showing the dense wrinkles on the extended and upfolded field. (**C**,**F**,**I**) Magnification of (**B**,**E**,**H**), respectively, showing thin fibers and laths on wrinkles. Arrows show position of enlarged areas.

In addition, the new materials of *Eohalobia* sclerites coexisting with the *Ocruranus* sclerites in the same rock samples are also show secondary phosphatization. The upfolded proximal margin at the apex of *Eohalobia* sclerites is a distinctive character, which forms an obtuse angle of approximately 90–120 degrees in a plane that is approximately perpendicular to the lateral plane and distal shell margin (Figure 2C,F). The length of *Eohalobia* sclerites and the extended and upfolded field (euf, Figure 2M) vary from 1.8–3.0 mm and 0.3–1.0 mm, respectively, and the width of the extended and upfolded field (=width of *Eohalobia* sclerites) varies from 1.3–2.7 mm (Table 1). The extended field in different specimens generally have similar shell widths, but their lengths vary greatly. For example, in *Eohalobia* sclerites with a similar width (approximately 2.0 mm) (CUBar70–25, CUBar110–7, CUBar228–5, CUBar110–2), the length of the extended and upfolded field shows great variation (0.3–0.6 mm) (Table 1). The parallel dense wrinkles and the varied length sizes of the extended field indicate that this part may be a retractable and weakly mineralized structure that may have a connecting function (Figure 2 and Figure 4B,E,H; Table 1). Moreover, the commonly broken extended proximal margin of these specimens could also be attributed to their original weak mineralization.

**Figure 5:**
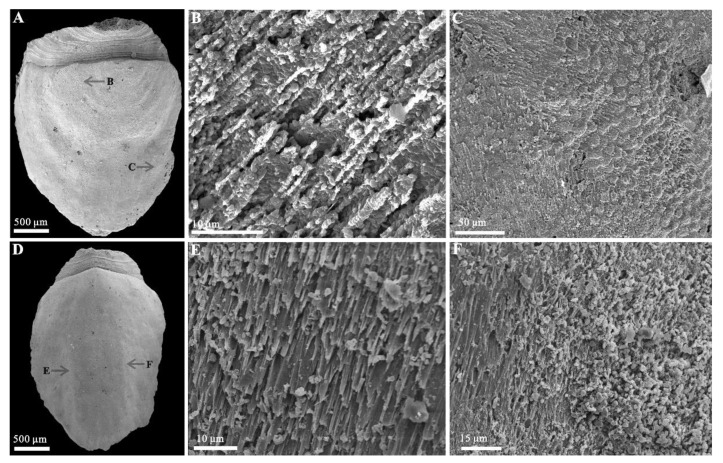
Lamello-fibrillar structure of *Eohalobia* sclerites. (**A**–**C**), (**D**–**F**) Internal molds, CUBar47–28, CUBar132–2, respectively. (**B**,**E**) Magnification of (**A**,**D**), respectively, showing the shape and size of fibers, which are horizontal and parallel to each other. (**C**,**F**) Magnification of (**A**,**D**), respectively, showing the fibers and convex polygons structure co-occurring on the surface of internal molds and gradually fused to lamellar units. Arrows show position of enlarged areas.

**Figure 6:**
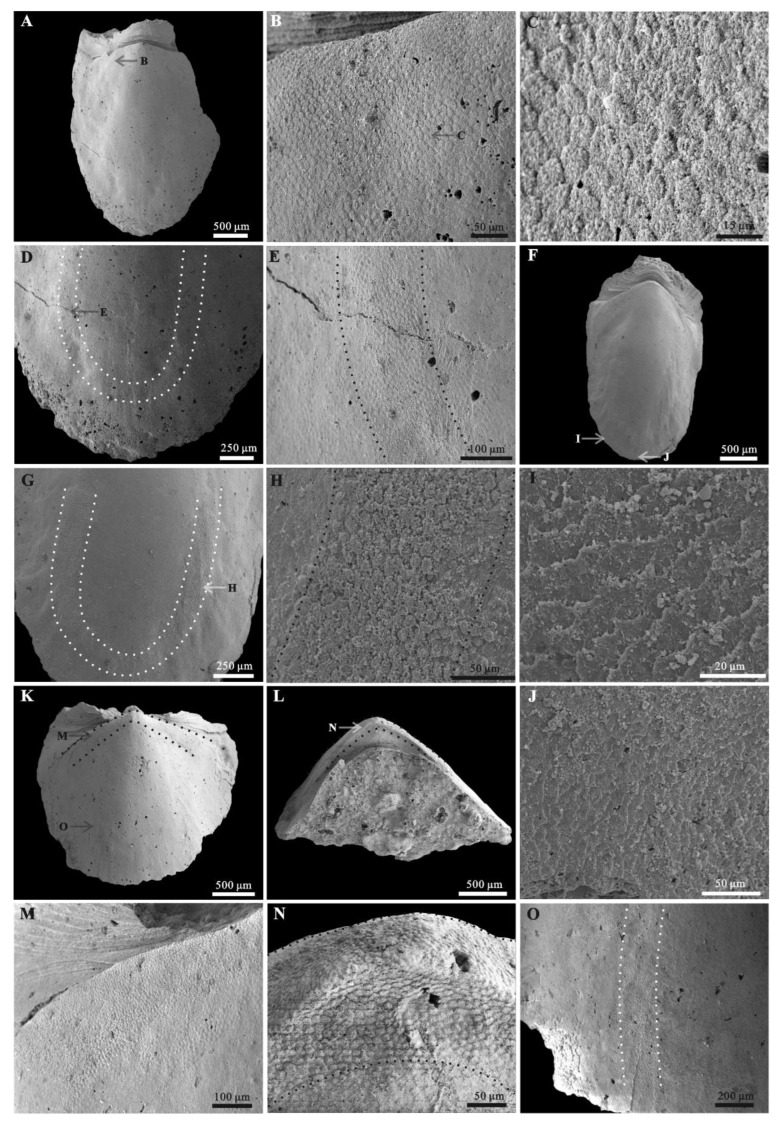
Muscle attachment zones and stepwise texture on *Eohalobia* sclerites. (**A**–**E**), (**F**–**J**), (**K**–**O**) Internal molds, CUBar121–2, CUBar132–3, CUBar227–6, respectively. (**A**–**K**,**J**,**M**,**O**) Dorsal view, (**L**,**N**) proximal view. (**B**,**M**,**N**) Magnification of (**A**,**K**,**L**), respectively, showing the distribution of muscle attachment zone in high-arched apical field sloping toward lateral margins. (**C**) Magnification of (**B**), showing the shape and size of convex polygonal texture. (**D**,**G**,**O**) Magnification of (**A**,**F**,**K**), respectively, showing the U-shaped muscle attachment zone symmetrically arranged on both sides of the middle surface near the lateral margins and interface between the muscle scar region and the rest of shell interior. (**I**,**J**) Magnification of (**F**), showing the stepwise texture occurred in the posterior and lateral margin. Arrows show the position of enlarged areas. Dotted lines zones show the distributions of muscle attachment zone.

The upfolded proximal margin (=the extended and upfolded field) of *Eohalobia* sclerites in previously reported materials seemed to be extremely short (see Figure 70 in [12]). However, the new material of *Eohalobia* sclerites illustrated herein show a much more extended proximal margin (=extended and upfolded field (euf)) (Figure 2, Figure 4A,D,G, Figure 5A,D and Figure 6A,F,K). Both lateral margins of the shell converge on an arched apex (ap) (Figure 2C,F,M and Figure 6L). An oblique cavity (oc) is formed between the initiation of the extended field and arched apex (Figure 2F and Figure 6L). The extended field folds up and breaks at a point with the same height of the sclerite that is opposite to the shell’s apex (Figure 2E). The extended field exhibits a high-arched central part (hcp) with two stretched lateral sides and forms the butterfly-shaped overall outline (Figure 2M and Figure 4G). It is noteworthy that the surface of the wrinkles exhibits a mineralized structure, including the thin fibrillar and laths (oriented, parallel to the shell surface) (Figure 4C,F,I).

### 3.2. Microstructures of Eohalobia Sclerites

Most early Cambrian mollusks are preserved as secondarily phosphatized shells or molds with few individuals bearing some fine imprints of the shell microstructure [26,33,34,35,36,37,38,39,40,41,42,43]. Although some of the early Cambrian SSFs are problematic due to incomplete preservation or bizarre morphology, the shell microstructure may help reveal their phylogenetic relationships [37,42,43]. The microstructure on the surface of the internal molds of *Eohalobia* in our collection reflects two types of inner shell microstructures: the lamello-fibrillar structure and the stepwise texture. Nevertheless, there are no microstructures observed in our new materials of *Ocruranus* sclerites from Stage 2. This may be due to abrasion during preservation or acid corrosion.

The fibers (less than 1 μm in diameter) of the lamello-fibrillar structure generally extend in different orientations in successive laminae (Figure 5B,C,E,F). This structure is one of the most common and basic microstructures in early Cambrian mollusks (see Table 1 in [37]) and may represent a lower control over biomineralization by the animal [39]. The lamello-fibrillar is typically inferred to be an aragonitic shell microstructure based on fibrous crystals [37,41,42]. This microstructure is similar to foliated aragonite in the modern gastropod *Lottia digitalis* (see Figure 4.3 in [39] and Figure 12.3 in [41]). It suggests that the new materials of *Eohalobia* sclerites in this study from Stage 2 and *Ocruranus* that occurred in the Fortunian both originally had a calcareous shell [26,30].

The stepwise texture occurs in areas near the lateral and distal margins (Figure 6F,I,J). It consists of calcareous units arranged in a stepwise pattern, each containing parallel transverse marks of fibers having serrate edges [36]. There are several different explanations for the formation mechanism of this stepwise pattern. In some mollusks, such as *Anabarella*, *Watsonella*, *Fordilla*, and *Pojetaia*, an oriented stepwise pattern at the apical and apertural part of the shell was interpreted as an inner layer consisting of fibers fused into partial well-developed lamellar units [33,43]. The stepwise texture may be closely related to certain laminar microstructures, especially of the lamello-fibrilla [35,36]. Moreover, the large nacre tablets may be considered an alternative explanation of the stepwise pattern [44]. In our material, fibers gradually transform into lamellar units with serrate edges (Figure 5C), which is consistent with that of *Watsonella* and *Pojetaia* [33,35,40]. Our study confirms that the stepwise texture and the lamella-fibrillar structure are closely related [40,45].

### 3.3. Muscle Attachment Zones on Eohalobia Sclerites

Convex polygonal microsculptures have been discovered on the surface of the internal molds of *Eohalobia* sclerites. The zones of these convex polygons are mostly distributed on the arched convex apical part sloping toward the lateral margin (Figure 6B,M,N) and on both sides of the middle surface near the lateral margins and distal margins, which seems to be U-shaped in symmetric and successive (Figure 6D,G). Each polygon is approximately 10 μm in diameter. There are distinct boundaries between them and those without central tubercles (Figure 6C,N). A similar microsculpture has been commonly discovered from the early Cambrian Mollusca [35,38,39,41,45,46,47,48].

There have been different interpretations of the polygons with varied shapes described in Mollusca [43]. Kouchinsky [36] suggested that the convex polygons (with a positive relief) may be due to the decalcification of prism-like units, whereas the concave polygons (with a negative relief) are possibly the result of preferential decay of organic sheets surrounding mineral prisms. Ushatinskaya and Parkhaev [38] interpreted the convex hexagons as the fossilized internal contents of cells in the muscle field area of *Eothele tubulus*, and the continuous concave polygonal netlike structure as cell imprints of the mantle epithelium. According to Vendrasco et al. [45], specifically in *Mellopegma*, the pits or concave polygons may represent a replacement or cast of the organic framework of a prismatic microstructure, with the pits representing the space where the prism crystals originated and grew. Moreover, the convex and concave polygonal textures were both interpreted as the imprints of muscle scars, for example, the polygonal microrelief structure in the columellar area of *Aldanella rozanovi* and the relief honeycomb microsculpture in the subapical area of *Oelandiella* sp. [46,47,48]. In this study, we prefer to interpret the zone with the imprints of the polygonal structures on the internal molds of the *Eohalobia* sclerites as the muscle attachment zone. Firstly, these simple polygons with almost the same size are convex and have a restricted distribution in a few stationary positions (Figure 6D,G,K,L). Secondly, compared with the prismatic shell microstructure and inter-prismatic membrane in a living unidentified oyster molluscan, the polygonal texture is more like the adductor muscle attachment zone of the living oyster (see Figure 6D in [43]).

## 4. Discussion

### 4.1. Ocruranus and Eohalobia Belong to the Same Scleritome

Qian and Bengtson [12] taxonomically revised sclerites of *Ocruranus* and assigned them into three species: *Ocruranus finial* Liu [19], *O. subpentaedrus* Jiang [17], and *O. trulliformis* Jiang [17]. The main differences between them are the morphologies of the sub-apical field and the apex. The sub-apical field of *O. finial* projects further at the midline; *O. subpentaedrus* has a straight or emarginate sub-apical margin, whereas *O. final* has a more protruding sub-apical margin (or apical re-entrant [26]); *O*. *trulliformis* has a prominent acute high apex compared with *O. final* and *O. subpentaedrus*. Recently, *O*. *trulliformis* has been considered as a helcionelloid due to its much taller apex and absence of the sub-apical region [26,27]. According to the before-mentioned description of morphological characteristics and size, our specimens of *Ocruranus* can be assigned to *O. subpentaedrus*.

*Eohalobia diandongensis* Jiang, 1982 was first reported from the Zhongyicun Member of the Zhujiaqing Formation in eastern Yunnan [18]. Qian and Bengtson [12] considered *Meishucunchiton* Yu, 1987, and *Cremnodinotus* Liu, 1987, as junior synonyms of *Eohalobia*, and based on their morphological similarity (the subapical field, the convex central part, and the re-entrant) and their co-occurrences, they assigned *Eohalobia* and *Ocruranus* to one fossil group. Vendrasco et al. [26] suggested that *Ocruranus* and *Eohalobia* belong to the same scleritome of an enigmatic organism and took *Eohalobia* as a junior synonym of *Ocruranus*. However, in this study, we continue to differentiate ‘*Eohalobia* sclerites’ and ‘*Ocruranus* sclerites’.

**Figure 7:**
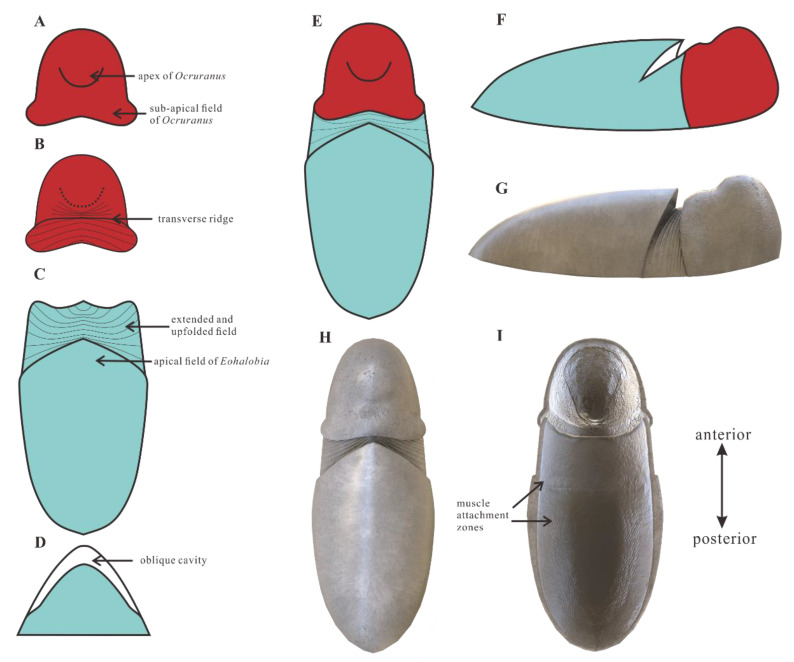
Two and three-dimensional reconstructions of *Ocruranus*–*Eohalobia* group. (**A**) Exterior view of *Ocruranus* sclerite, showing the apex and sub-field of *Ocruranus* sclerite. (**B**) Interior view of *Ocruranus* sclerite, showing the transverse ridge. (**C**) Dorsal view of *Eohalobia* sclerite, showing apical field, extended and upfolded field (weakly mineralized structure) of *Eohalobia* sclerite. (**D**) Proximal view of *Eohalobia* sclerite, showing the oblique cavity of *Eohalobia* sclerite. (**E**) Dorsal view of the complete profile of *Ocruranus*–*Eohalobia* group. (**F**) The lateral view of the complete profile of *Ocruranus*–*Eohalobia* group. (**G**–**I**) Lateral, dorsal, and ventral view of three-dimensional *Ocruranus*–*Eohalobia* group, respectively, (**I**) showing the distributions of muscle attachment zones.

According to observations made on the new material from the Yanjiahe Formation (Cambrian Stage 2), *Ocruranus* and *Eohalobia* sclerites are preserved as cast and steinkern, respectively. The reason for this difference in preservation is still unsolved. One possible reason is that the original shell of *Eohalobia* was relatively thinner than that of *Ocruranus* and the elongated shell is more easily crushed during preservation. The morphological characteristics of *Ocruranus* and *Eohalobia* sclerites from the Fortunian and Stage 2 do however show some differences. For example, the extended and upfolded proximal field on *Eohalobia* sclerites from the Member 5 of the Yanjiahe Formation is reported here first. As parts of the scleritome of a multi-plated organism, the isolated sclerites are commonly slightly variable in shape. However, the similarity of some characteristic morphologies (the arched and elongated shell with an arched proximal/apical margin) indicate that the new material from Cambrian Stage 2 can certainly be assigned to *Eohalobia diandongensis*. There is considerable evidence to support that *Ocruranus* sclerites and *Eohalobia* sclerites are two parts of the same skeleton in our new material. (1) The quantity of *Ocruranus* sclerites and *Eohalobia* sclerites co-occurring in Member 5 of the Yanjiahe Formation is roughly equal to the ratio of 1:1.6. (2) Although the *Eohalobia* sclerites tend to be more elongated, its extended fields of *Eohalobia* sclerites are consistent with the cast specimens of *Ocruranus* sclerites in width. (3) Both sclerites of *Ocruranus* and *Eohalobia* from the Yanjiahe Formation have the same butterfly-shaped extended field and have dense wrinkles (Figure 2M and Figure 3B,C,E,F). (4) The size range of both sclerites is consistent. (5) The wrinkles on both of the two types of sclerites display a parallel distribution and are bent toward a high point (Figure 2M and Figure 3C). (6) The extended field of *Eohalobia* sclerites (Figure 4) and the inner surface of *Ocruranus* sclerites in the new material match the morphology and ornament (Figure 3B,C,E,F), which suggests that *Ocruranus* should partly overlie *Eohalobia* (Figure 7E).

The disputes about the anterior and posterior orientation of the *Ocruranus*–*Eohalobia* scleritome in previous studies remains to be solved. In living chitons, the tail valve generally exhibiting an articulamentum layer, or a layer composing the insertion plates and the sutural laminae of a valve, projects into the girdle and underlies the preceding valves in series [49]. *Eohalobia* also bears comparable two layers, the lower extended and upfolded field and the upper shell. That is to say, the extended field of *Eohalobia* sclerites underlies the *Ocruranus* sclerites. Thus, the extended and upfolded field of *Eohalobia* sclerites (Figure 7C) and the extended sub-apical field of cast specimens of *Ocruranus* sclerites (Figure 7B) are both possible articulating projections. The *Eohalobia* and *Ocruranus* sclerites described above might be the tail valve and the head valve respectively in our scleritome reconstruction. It is worth noting that the broken edge of the extended field of *Eohalobia* sclerites is upward lifting, implying that the *Ocruranus* sclerites are not located at the same height as the *Eohalobia* sclerites.

Although the antero-posterior axis and joint types can be outlined, the number of valves in the reconstruction of the original scleritome of *Ocruranus*–*Eohalobia* is still controversial. Vendrasco et al. [26] reconstructed the skeleton of the *Ocruranus*–*Eohalobia* group and hypothesized that the *Ocruranus subpentaedrus*–*Eohalobia diandongensis* group included three types of valves and the number of valves was possibly three or eight. Herein, we consider that the Fortunian *Ocruranus subpentaedrus*–*Eohalobia diandongensis* group only has three valves with *Ocruranus subpentaedrus* and *Eohalobia diandongensis* as two end valves and *Gotlandochiton*? *minimus* as the only intermediate valve. Firstly, there is no strong evidence to show that they have an overlapped jointing structure. Besides, if there are enough specimens, it is possible to determine the number of intermediate valves by counting the specimen of head valves, intermediate valves, and tail valves collected in the same member or locality [50]. However, the quantity of the three types of valves was not accounted for in Vendrasco et al. [26].

Although the possible isolated intermediate valves (*Gotlandochiton*? *minimus*) are yet to be found in Member 5 of the Yanjiahe Formation, a single unique internal mold specimen of a *Eohalobia* sclerite shows a butterfly-shaped part forming a V-shape with a transverse furrow between the broken anterior edge and the apex of the shell (Figure 3G,H,I). This butterfly-shaped part corresponds with the extended field of *Eohalobia* sclerites, the sub-apical field of *Ocruranus* sclerites and *Gotlandochiton*? *minimus* in shape. This unique specimen is supposed to be a juvenile sclerite, considering its much smaller size compared with the majority of co-occurring sclerites of *Eohalobia* and *Ocruranus* (1 mm in width and 1.5 mm in length of the special specimen, whereas the average width and length of *Eohalobia* sclerites are 2 mm and 2.7 mm). To explain the difference between this unique specimen at the juvenile stage and other *Eohalobia* sclerites at the adult stage, we speculate that: the butterfly-shaped part is corresponding with the extended and upfolded field in adult *Eohalobia* sclerites; as it grows, the transverse furrow (Figure 3G) gradually extends to both sides of the lateral margin. The dense wrinkles were not observed in the butterfly-shaped part, which may be a result of preservation variations or a lack of development at the juvenile stage. Thus, the new material from Member 5 of the Yanjiahe Formation (Stage 2) presented in this paper shows that the adult *Ocruranus subpentaedrus*–*Eohalobia diandongensis* group has only two overlapping valves.

According to the description and analysis of the characteristics of the new materials of *Ocruranus* sclerites and *Eohalobia* sclerites from Member 5 of the Yanjiahe Formation, we propose a hypothetical reconstruction of the scleritome of the *Ocruranus*–*Eohalobia* group (Figure 7). The difference in the number of plates in the *Ocruranus subpentaedrus*–*Eohalobia diandongensis* group scleritome in the Fortunian (three plates) and Stage 2 (two plates) of the Cambrian may result from morphological evolution or other factors (geographical, biological, etc.).

### 4.2. Zoological Affinity

Vendrasco et al. [26] mainly assigned the *Ocruranus*–*Eohalobia* group to the polyplacophoran molluscs, according to a range of morphological characteristics (e.g., the single row of the overlapping arched shell plates and the rounded anterior projections of *Ocruranus*) and the aragonitic shell. In our new materials, besides the adjacent valves overlaying in sequence in the reconstruction of *Ocruranus*–*Eohalobia* scleritome being consistent with modern chitons, the distributions of muscle attachments may provide new evidence about the zoological affinity of the *Ocruranus*–*Eohalobia* group.

In *Chelodes* (silicified chitons reported from the Upper Wenlock (Silurian) of Gotland, Sweden), the separated transverse muscles articulate adjacent sclerites between the apical area and anterior marginal furrow (see Figure 4 in [51]). The distribution of these transverse muscles in the *Ocruranus*–*Eohalobia* group is consistent with *Chelodes*. Furthermore, the similar structures of fine myofibrils of the prototroch ring in the early larval stages of modern chitons (see Figure 2 in [52]) also existed in the apical area of *Eohalobia*. Thus, like living chitons, *Eohalobia* sclerites and *Ocruranus* sclerites may also articulate to each other by the muscle bundles on the arched convex apical part, which indicates that the *Ocruranus*–*Eohalobia* group probably belongs to the stem lineage of chitons, as suggested by Vendrasco et al. [26]. Moreover, the similar distribution of transverse muscles between the tail valve and the preceding valve in both modern chitons ([52], see Figure 20.4D in [53]) and *Eohalobia* also supports this hypothesis. In addition, the U-shaped muscle attachment zone distributed on the middle surface of *Eohalobia* (Figure 5E,H) also corresponds to the position of posterior insertion of longitudinal and lateral muscles in modern chitons (see Figure 20.4D in [53]; plate XXXI in [54]). Cherns et al. [51] also suggested that the transverse and lateral longitudinal muscles on later sclerites facilitate intimate attachment between adjacent sclerites. It is worth noting that the longitudinal and lateral muscles in modern chitons are commonly linked with the transverse muscle, whereas the U-shaped muscle attachment zone is isolated with the muscle in the arched convex apical part in *Eohalobia* sclerites. This difference may be caused by preservation. Moreover, the similar pattern of the U-shaped muscle scar arrangement in Monoplacophora (see Figure 20.3D in [53]) and Polyplacophora may reflect that such an arrangement is an efficient way for a bilaterally symmetrical lophotrochozoans to be able to pull their shell plates down and keep the tight attachment between the shell and the soft tissues.

Due to the limitation of fossil preservation, we tentatively interpret the zoological affinity of the *Ocruranus*–*Eohalobia* group by comparing the distribution of the muscle attachment zones with that of modern chitons. The muscle attachment zones distributed in the arched convex apical part and the middle surface of *Eohalobia* sclerites and the configuration of sclerites show strong evidence that the *Ocruranus*–*Eohalobia* group can be assigned to a chiton-like mollusc. As a possible chiton-like form before the split between aplacophorans and chitons, the *Ocruranus*–*Eohalobia* group from the early Cambrian may provide significant information for the origin of the aculiferans, which aplacophorans are probably derived from with their chiton-like forms [31].

### 4.3. Comparison with Early Palaeozoic Polyplacophorans

**Figure 8:**
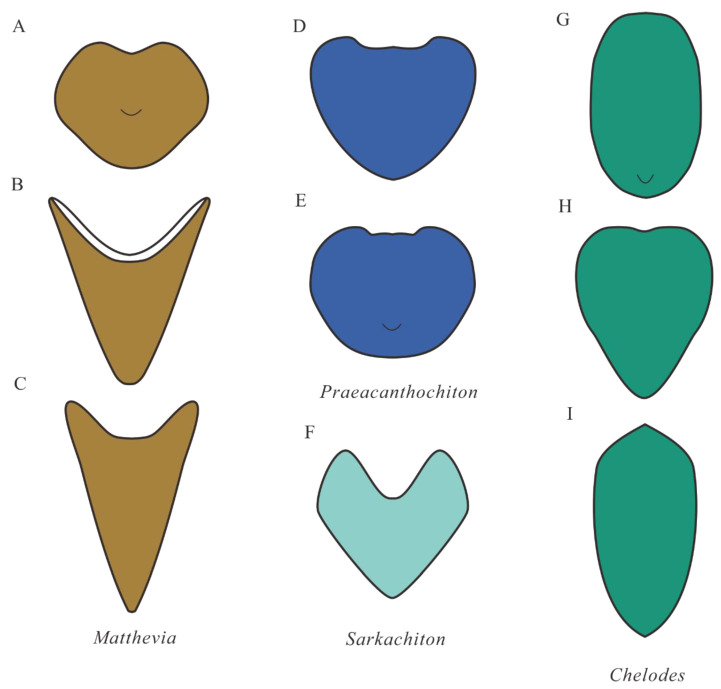
Simple outline of early Palaeozoic Polyplacophorans. (**A**–**C**) *Matthevia*, dorsal view, head valve, intermediate valve, and tail valve, respectively (modified from explanation of plate 1 in Runnegar et al. [50]). (**D**,**E**) *Preacanthochiton*, dorsal view, intermediate valve, and tail valve, respectively (modified from explanation of plate 2 in Runnegar et al. [50]). (**F**) *Sarkachiton*, dorsal view, intermediate valve (modified from plate 53 in Dzik [64]). (**G**–**I**) *Chelodes*, dorsal view, head valve, intermediate valve, and tail valve, respectively (modified from text-Figure 4 in Cherns [58], Figure 2 in Cherns [51], and explanation of plate 2 in Runnegar et al. [50]).

Comparing the arrangement of the sclerites of the *Ocruranus*–*Eohalobia* group with that of other early Palaeozoic polyplacophorans (e.g., *Matthevia*, *Praeacanthochiton, Sarkachiton,* and *Chelodes* in Figure 8) may provide important data for the evolution of polyplacophorans. *Matthevia* from the late Cambrian was established as a polyplacophoran by Runnegar et al. [50], and it may possess one subcircular and flattened head valve, five tall and conical intermediate valves, and one laterally compressed conical tail valve. The head valve of *Matthevia variabilis* is similar to *Ocruranus* in cap-like shape, but the size of *M. variabilis* (length approximately 1 cm) is larger than *Ocruranus* (length approximately 600 μm). The morphology of *M. variabilis* further departs from that of *Ocruranus* in the absence of the sub-apical field. The intermediate valves of *M. variabilis* are similar to that of *Eohalobia* with the elongated shell and the reversed V-shaped cross section in anterior view, but the apparent differences are that the former has a subconical shape and a barbed arrowhead-shaped posterior margin (see the description of *Matthevia wahwahensis* in [55]). Moreover, the intermediate valves of *M. variabilis* possess a conical cavity in ventral view and a concave outline in the middle part of the anterior margin. The tail valves of *M. variabilis* have no similarity with *Ocruranus* and *Eohalobia.*

*Preacanthochiton* from the Lower Ordovician was a tiny, apparently advanced chiton [50]. No convincing head valve of this genus was ever found. The intermediate valves of *Preacanthochiton* are slightly elongated with a flat posterior surface connecting the adjacent valves. As described by Runnegar et al. [50], each probable tail valve has a subcentral apex and an angular bend at the symmetry plane with the microornament of fine granules. Indeed, although the tiny size of *Preacanthochiton* is similar to that of *Ocruranus* and *Eohalobia*, the most obvious difference between them is the occurrence of numerous fine granules on the external surface of *Preacanthochiton*.

*Sarkachiton* was a new genus revised by Dzik [56], as a typical Septemchitonidae, with a strong V-shaped cross section. The head valve of *Sarkachiton* was not found and only given a conjectural shape in the reconstruction of the whole armor ([56]; Figure 14C in Dzik, [57]). The short and thick intermediate valves show an acute triangle posterior margin and a concave middle part at the anterior margin with a concave middle part. The prominent tubercles pointing backward in the intermediate valves are distinct from *Ocruranus* and *Eohalobia*. The probable tail valve of *Sarkachiton* is more elongated than the intermediate valve. The inner surface of the tail valve has a conical cavity penetrating to its apex, which is extremely similar to that of *Matthevia*.

*Chelodes*, as a less disputed chiton, was a broadly defined genus of high morphological variability that had a long stratigraphical range (Lower Ordovician to upper Silurian/Lower Devonian) and a wide geographical distribution [50,55,58]. The head valve of *Chelodes* is generally similar to the intermediate valve but with a rounded anterior margin. The elongated tail shell is somewhat similar to *Eohalobia* sclerite but without the convex apex. The intermediate valves of *Chelodes* were extensively reported and show the acute triangle posterior part with one-third to one-half of the entire shell length and the deeply embayed anterior margin in dorsal view. The ventral surface of the intermediate valves of *Chelodes* exhibits the conical cavity below the margin of the flat posterior face, which is obviously different from the situation in both *Ocruranus* and *Eohalobia*. The tail valve with an elongated shell and convex apex in the anterior margin is quite similar to *Eohalobia* (especially figs. 54–56 of plate 2 in [50]), except for the size. Moreover, all valves of *Chelodes* are externally covered by comarginal growth lines.

Except for the above-mentioned genera *Matthevia* Walcott, 1885, *Sarkachiton* Dizk, 1994, *Preacanthochiton* Bergenhayn, 1960, *Chelodes* Davidson and King, 1874, polyplacophorans are widely reported in the early Palaeozoic, for example, *Hemithecella*, *Eukteanochiton, Orthriochiton* [50,51,55,56,57,58,59,60,61,62,63,64,65]. The reconstruction and orientation of these early Palaeozoic polyplacophorans are mostly based on the morphology and structure of the isolated valves and the comparison of the arrangement of shell plates with living chitons. If the *Ocruranus*–*Eohalobia* group corresponds to ancestral chitons, this work may provide significant information for studying the origin and early evolution of polyplacophorans.

## 5. Conclusions

The material from Member 5 of the Yanjiahe Formation (Cambrian Stage 2) provides new insights into the morphological characteristics of the *Ocruranus*–*Eohalobia* group, such as wrinkles, the muscle attachment zone, and shell microstructures. The extended and upfolded fields of the *Eohalobia* sclerites exhibit dense wrinkles and have quite varied lengths, which possibly suggests that this part is a flexible and weakly-mineralized structure with connecting function. The wrinkles exist on both *Eohalobia* and *Ocruranus* sclerites and are extremely similar in appearance, implying that these two types of valves should be buckled together. One unique specimen suggests a possible growth process from the juvenile to adult stage. The distribution of the muscle attachment zones of the *Eohalobia* sclerites is comparable to recent chitons. *Ocruranus* and *Eohalobia* sclerites may respectively correspond to the head and tail valves of the same scleritome in an overlapping arrangement, which implies that the *Ocruranus*–*Eohalobia* group is most probably assigned to a chiton-like molluscan animal.

## Figures and Tables

**Table 1:** Measurements of 12 well-preserved specimens of *Eohalobia* sclerites.

Specimens Number	Length of Extended and Upfolded Field (mm)	Width of Extended and Upfolded Field (=Width of *Eohalobia* Sclerites) (mm)	Length of *Eohalobia* Sclerites (mm)
CUBar70–25	0.300	2.083	2.951
CUBar121–7	0.319	1.605	-
CUBar110–7	0.318	2.053	2.220
CUBar228–5	0.430	2.046	2.339
CUBar212–1	0.554	1.900	2.579
CUBar227–3	0.531	1.349	1.840
CUBar47–28	0.536	2.422	3.075
CUBar132–3	0.657	1.588	2.655
CUBar126–12	0.589	2.062	2.622
CUBar121–11	0.650	2.697	3.643
CUBar40–1	0.841	2.153	2.824
CUBar212–2	1.027	1.817	3.003

## Data Availability

Not applicable.
